# All-fiber continuous-wave Raman fiber oscillator operating at 2118 nm

**DOI:** 10.1038/s41598-019-44502-x

**Published:** 2019-06-03

**Authors:** Zhao Quan, Jianhua Wang, Hui Shen, Xiaolong Chen, Yunfeng Qi, Bing He, Jun Zhou

**Affiliations:** 1 Shanghai Key Laboratory of All Solid-State Laser and Applied Techniques, Shanghai Institute of Optics and Fine Mechanics, Chinese Academy of Sciences, Shanghai, 201800 China; 2 Space Energy University, Beijing, 101416 China

**Keywords:** Mid-infrared photonics, Nonlinear optics, Fibre lasers

## Abstract

An all-fiber Raman fiber oscillator operating at 2118 nm pumped by homemade thulium-doped fiber laser with continuous output power of 4.2 W has been demonstrated in this paper. By means of 26 m ultrahigh-numerical-aperture fibers, the average slope efficiency can be up to 23.6%. Because of the Raman spectrum broadening of the first Stokes light, the intensity of high order Raman Stokes has been suppressed. The mechanism of Raman spectrum broadening has been analyzed by group-velocity dispersion and Four-Wave Mixing frequency theories in detail. Two mode field adaptors have been used to reduce the intracavity loss and thermal deposition, which indicates that the proposed architecture is robust and has significant power scaling potential.

## Introduction

Simple and robust laser sources operating at >2.1 μm wavelength are desirable for a wide range of applications including medicine, nonlinear optics and other fields. The prevalent method to acquire laser sources is stimulated emission of rare-earth-doped gain medium. For instance, a number of holmium-doped crystal^1^ and fiber^2–4^ lasers have been demonstrated to enable laser emission at >2.1 μm wavelength. However, as the requirements for laser move towards longer wavelength, these architectures are limited due to expensive cost and difficulties in obtaining gain materials.

Raman fiber laser has been listed as an interesting direction in mid-infrared laser region^5^, due to the flexibility of laser emission at almost any desired >2.1 μm wavelength by means of nonlinear wavelength conversion. Because the absorption losses of guiding light in traditional silica fibers are naturally large, researchers usually construct Raman lasers by pulse mode^6,7^. Yet the pump sources of pulse Raman lasers are complicated and the average powers are usually quite low. In 2004, single-mode Raman fiber lasers emitting a few hundreds of milliwatts at wavelength of 2.0 and 2.2 μm were demonstrated^8^. After that, plenty of continuous-wave (CW) Raman fiber laser systems have been established. For example, a Raman fiber amplifier at 2147 nm seeded by sophisticated Tm-doped fiber laser has been demonstrated with highly nonlinear fiber as gain fiber^9^. Although 3.7 W fluoride glass Raman fiber laser operating beyond 2.2 μm was reported^10^, mechanically weak soft glass fiber as host material shows significant limitations in practice. In 2007, CW Raman laser at 2.1 μm was demonstrated in GeO_2_ fiber based on free-space coupling^11^, which was inconvenient to integrate. Besides, the second-order Stokes was observed at 20 W pump level in ref.^11^, and there is still some room for improvement.

It is noted for a long time that the intrinsic infrared absorption of GeO_2_ glass and the impurity of the Ge-OH absorption bands are shifted to longer wavelength than those in silica glass^12^. Moreover, GeO_2_-doped fibers have physical properties similar to silica fibers, which means the design of all-fiber system is easy by splicing each other. To date, by using highly Ge-doped silica-based fiber, the longest wavelength of Raman laser is 2.43 μm with output power of 0.3 W has been obtained^7^.

In this paper, 4.2 W CW all-fiber Raman oscillator operating at 2118 nm is reported by means of 26 m ultrahigh-numerical-aperture (UHNA) fiber for the first time. Since the Raman gain is proportional to pump laser intensity, it is important to increase pump laser intensity by reducing intracavity losses. Two-way mode field adaptors (MFAs) have been used to reduce splice loss between fibers with different core diameters. Meanwhile, the proposed resonant cavity formed by a pair of fiber Bragg gratings (FBGs) can decrease the threshold of Raman fiber laser. The high-order Stokes light has been totally suppressed due to the first-order Stokes spectrum broadening. The average slope efficiency is achieved up to 23.6% with respect to incident pump light which is the highest record to the best of our knowledge.

## Results

**Figure 1:**
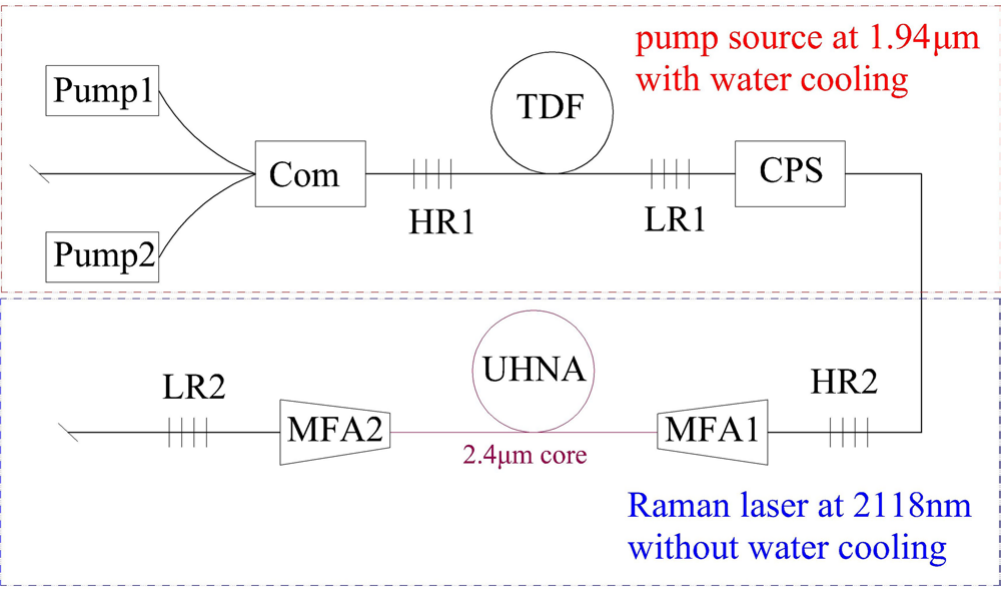
Schematic diagram of experimental setup. Pump1 and Pump2 were two LDs at 793 nm. Com was a (2 + 1) × 1 fiber combiner. HR1 and LR1 were high reflectivity and low reflectivity FBGs of 1.94 μm. TDF was Tm-doped fiber. CPS was cladding stripper. HR2 and LR2 were high reflectivity and low reflectivity FBGs of 2118 nm. UHNA was ultrahigh-numerical-aperture fiber. MFA1 and MFA2 were two-way mode field adaptors.

**Figure 2:**
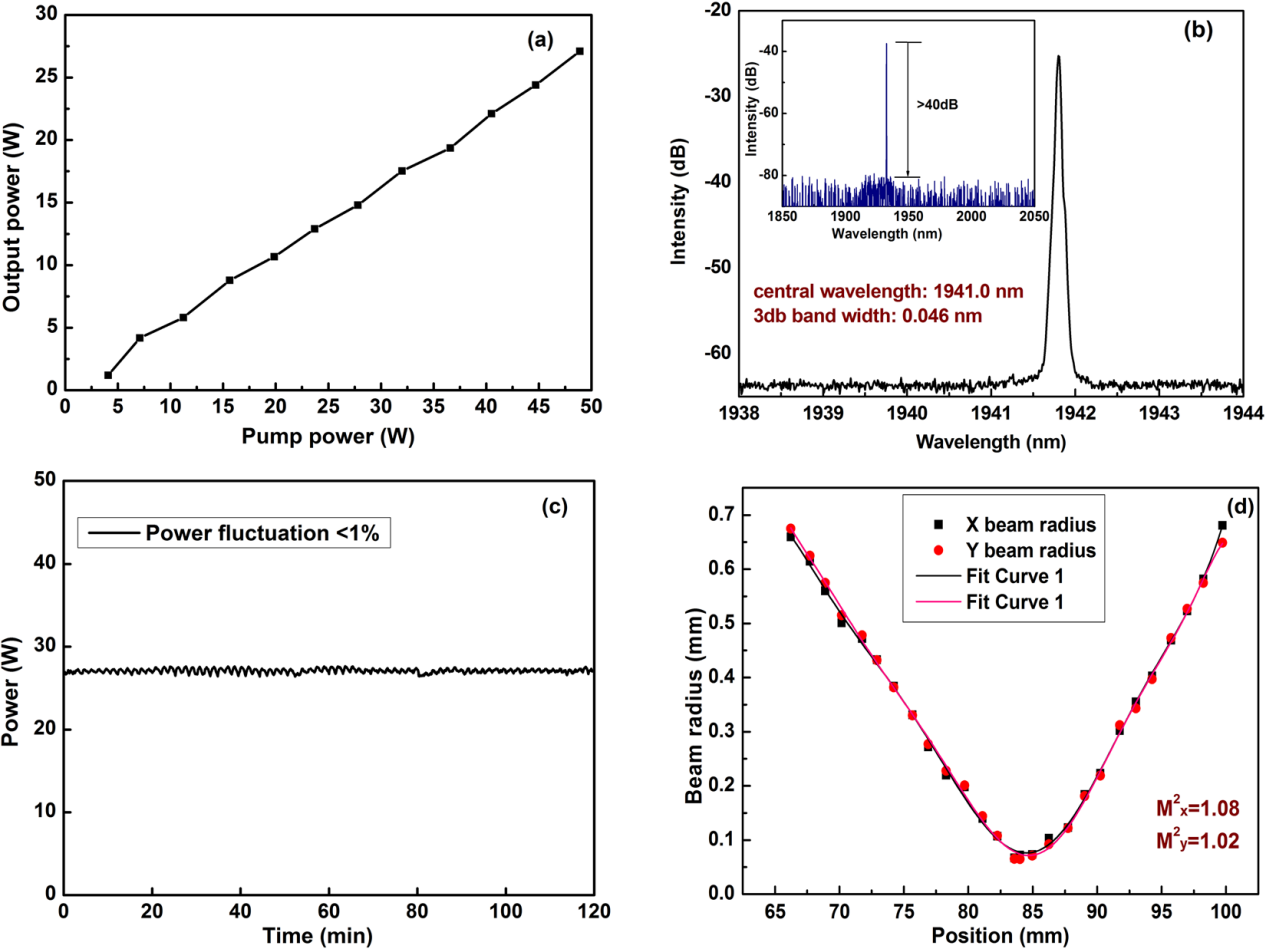
Laser output character of 1.94 μm pump source. (**a**) Evolution of output power as linear function of incident pump power at 793 nm; (**b**) optical spectrum with 3 dB bandwidth of 0.046 nm; (inset: wide spectrum of 200 nm with no ASE at over 40 dB); (**c**) power fluctuation of less than 1% in 2 hours; (**d**) near diffraction-limited beam quality with M^2^_x, y_ < 1.1.

**Figure 3:**
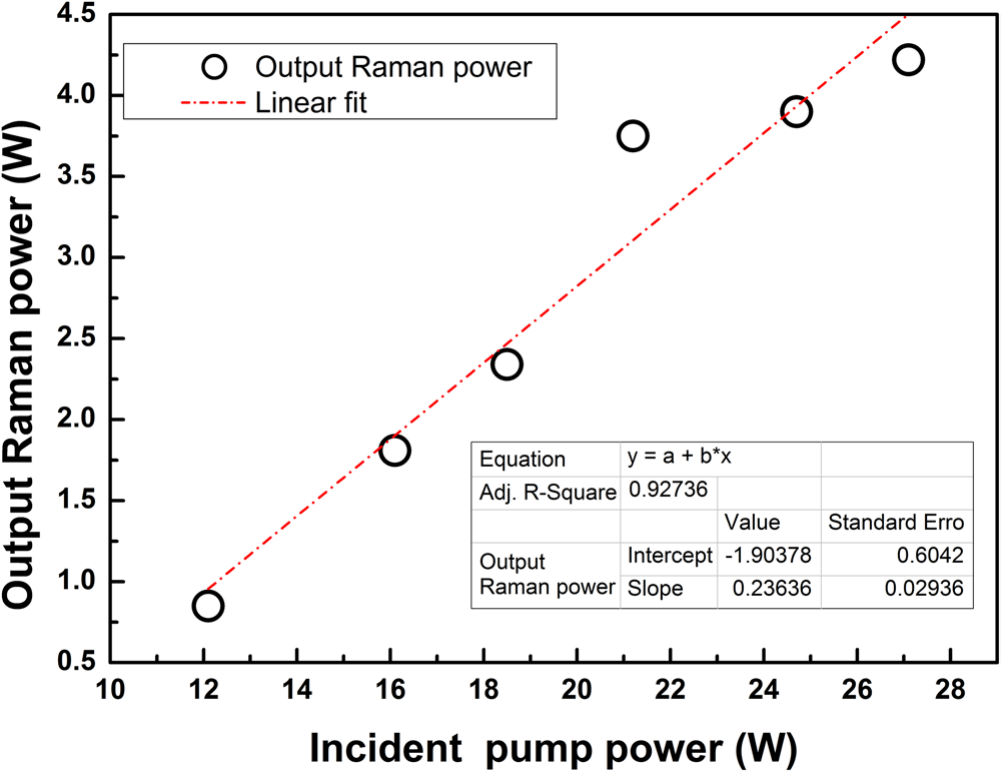
Evolution of output power at 2118 nm laser as linear function of incident pump powers at 1.94 μm.

Figure 1 shows that the continuous-wave Raman laser operating at ~2118 nm has been explored by simple and robust Raman oscillator. The incident pump power is obtained by a homemade 1.94 μm pump source as shown in Fig. 2. The output power of Raman laser is illustrated as function of incident pump power in Fig. 3. Maximum output power of 4.2 W at 1^st^ Raman stokes light is achieved with an average slope efficiency of 23.6% with respect to incident pump power.

**Figure 4:**
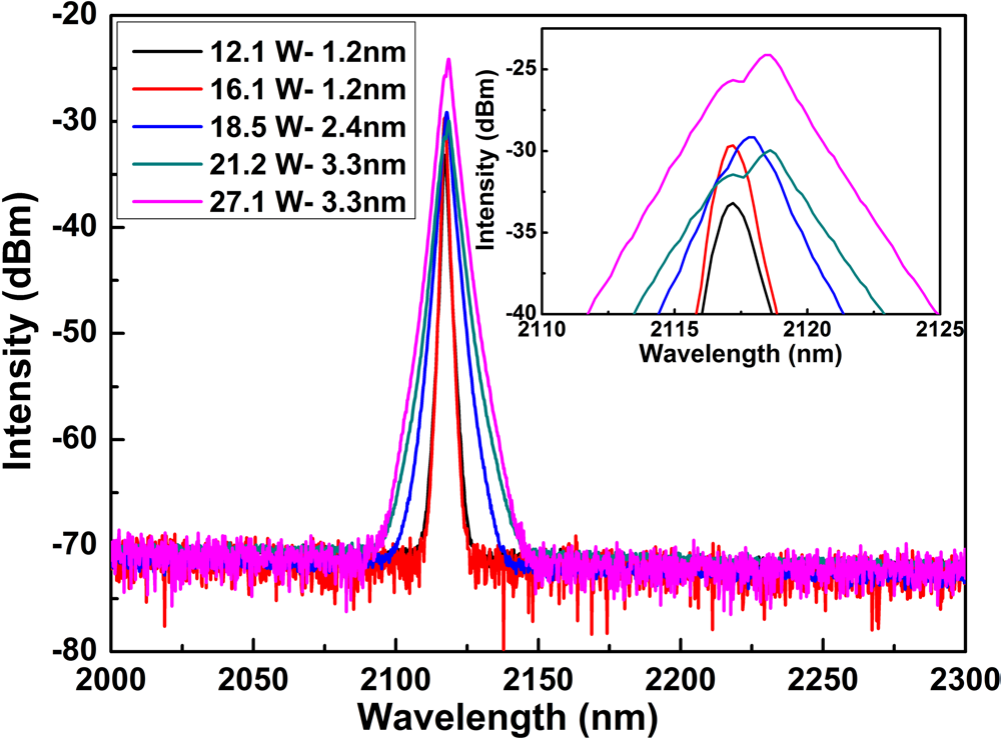
Output spectra and FWHM of the CW Raman laser at different incident pump power with resolution of 1 nm (inset: details of Raman peak wavelength).

**Figure 5:**
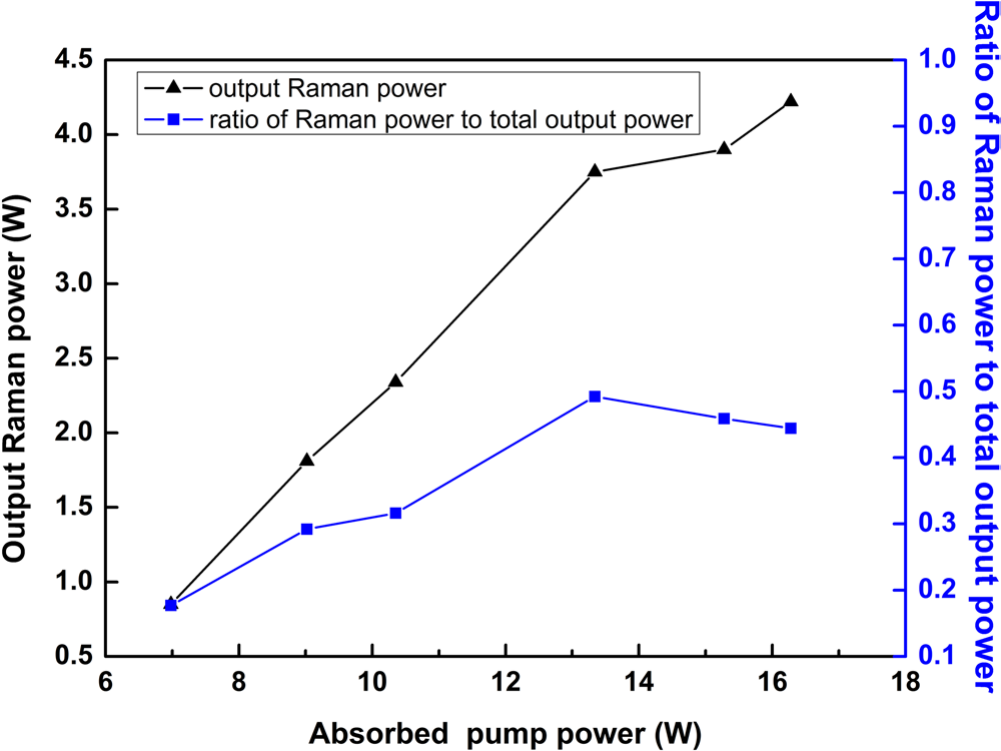
Relation between Raman power (black) with ratio of Raman power to total output power (blue) and incident pump power.

**Figure 6:**
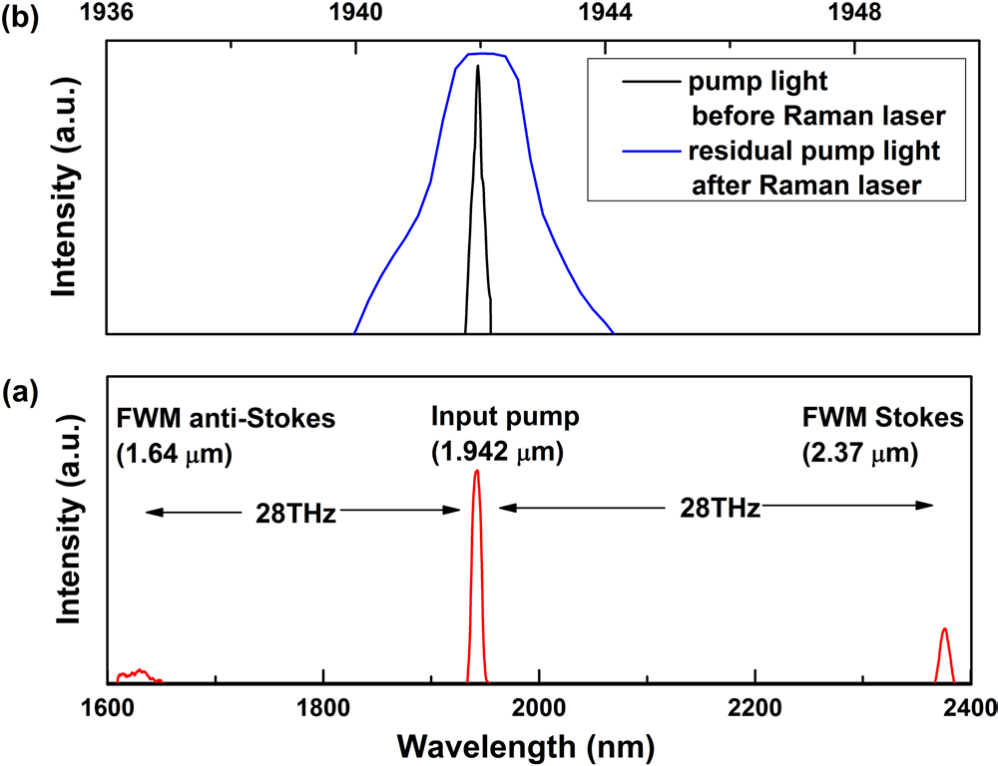
(**a**) Spectra of FWM Stokes and anti-Stokes peaks of pump wave below Raman gain threshold; (**b**) spectra of pump light at 1.94 μm before Raman laser and residual pump light after Raman laser.

**Figure 7:**
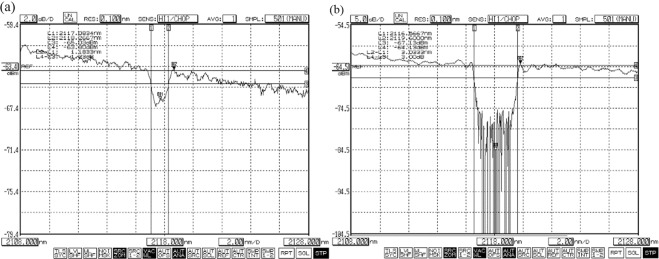
Transmission profiles of FBGs. (**a**) LR2 with bandwidth (FWHM) of 1.2 nm; (**b**) HR2 with bandwidth (FWHM) of 3.3 nm.

Output spectra of the CW Raman laser at different incident pump power are showed in Fig. 4. And the slightly red-shift of central wavelength is consistent with Raman power. Figure 5 shows the ratio of Raman laser power to total output power. The mechanism of spectral broadening and slightly red-shift of central wavelength are analyzed in detail through Figs 6 and 7 and considered to be related to Four- Wave Mixing (FWM).

**Figure 8:**
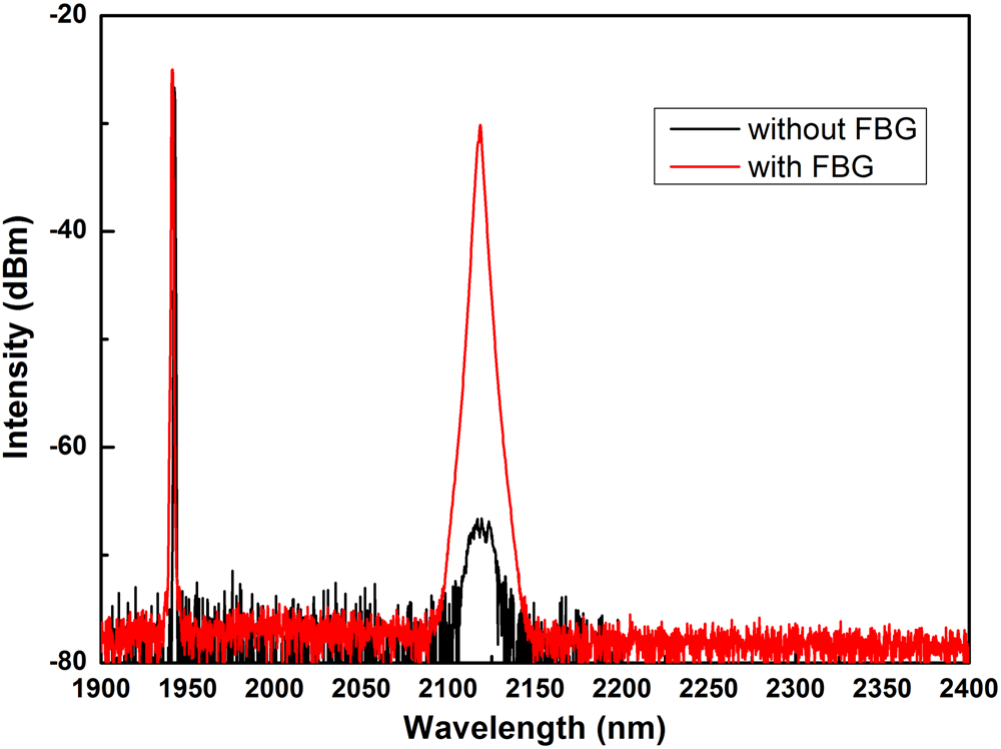
Laser output spectra of with or without FBG at 4.2 W Raman power.

Different spectra under two circumstances (with or without FBG) at 4.2 W Raman power are showed in Fig. 8. It shows that by adding FBGs as resonant cavity, the intensity of 1^st^ Raman Stokes laser is obviously enhanced and no high-order Stokes light is observed. The Raman gain is estimated to be 1.9 dB/(km·W), which will be increased by further working on system optimization.

**Figure 9:**
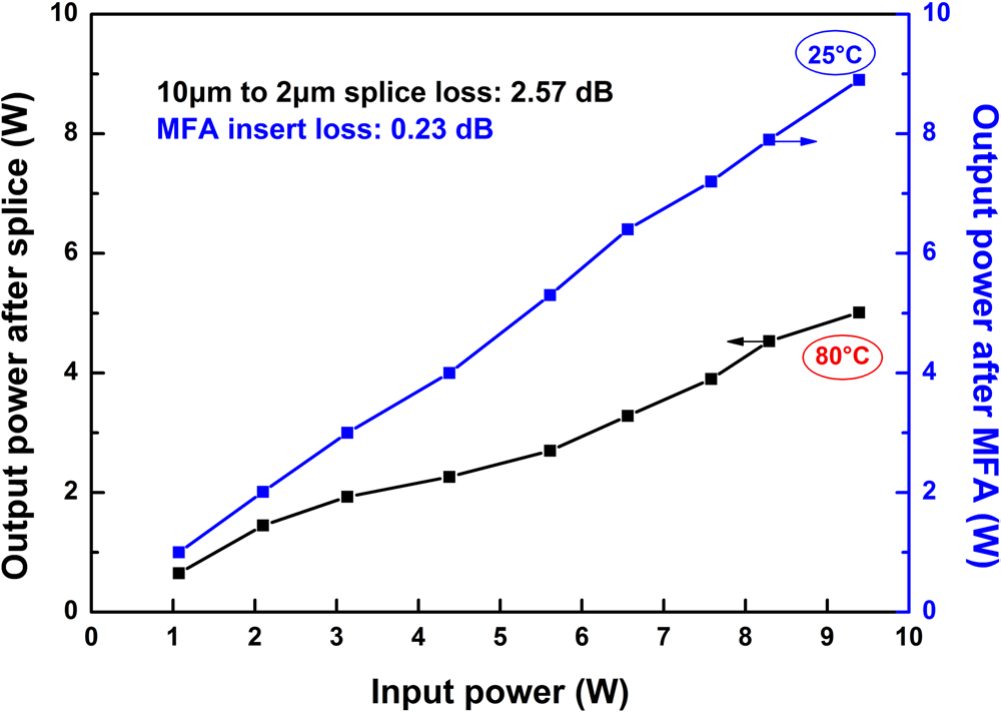
Output power after MFA and splice between 10 μm and 2 μm versus input power.

Two-way MFAs are used to replace fusing point between Raman gain fiber and other fibers. The differences of thermal deposition and system loss between MFAs and splice point are shown in Fig. 9. The results show that there is no thermal deposition in the case of MFAs and the power fluctuations is measured to be less than 2% in 0.5 hour.

## Discussion

Normally, a stable and reliable pump source is crucial to a Raman laser. In Fig. 2, the homemade pump source^13^ for Raman laser is single-mode CW fiber laser operating at 1941.8 nm with maximum output power of 27.1 W and near diffraction-limited beam quality. The output power increases linearly with the pump power is shown in Fig. 2(a), which indicates that the output power is only limited by the available pump power. Figure 2(b) shows the emission spectrum with narrow linewidth of 0.046 nm which offers high Raman conversion efficiency. Figure 2(c) shows the power fluctuation is measured less than 1% within 2 hours. The near diffraction-limited beam quality (M^2^_x,y_ < 1.1) is shown in Fig. 2(d). The proposed architecture provides robust and excellent pump source for subsequent Raman fiber laser.

Raman laser output power as function of incident pump power is illustrated in Fig. 3. The incident pump power is obtained by detecting homemade 1.94 μm pump source directly. The laser generates maximum Raman output power of 4.2 W, and reaches threshold at pump power of 12.1 W due to high splice loss and insertion loss of components in Raman laser system. The average slope efficiency is up to 23.6%. Moreover, the output power increases linearly with the incident pump power at low power levels with a higher slope efficiency, but begins to depress when pump power exceeds 21.2 W. It is suggested that this behavior is related to long length of Raman gain fiber, which leads to non-uniform pump deposition density distribution along the fiber^14^. Besides, the low slope efficiency may also indicate gain competition with other nonlinear effects as analyzed in the following.

Output spectra of the CW Raman laser at different incident pump power with central wavelength of ~2118 nm are shown in Fig. 4 with a resolution of 1 nm. At low pump power from 12.1 W to 16.1 W, only the intensity of Raman laser increases, while the width of Raman spectrum stays the same (FWHM of 1.2 nm, and span of 20 nm from ~2109 nm to 2129 nm). However, when pump power increases from 16.1 W to 21.2 W, the Raman laser intensity maintains stability. The FWHM of Raman laser obviously broadens to 3.3 nm instead, and the span broadens to 60 nm from ~2094 nm to ~2154 nm as well. At higher incident pump power of 21.2~27.1 W, the intensity of Raman laser is enhanced while the bandwidth stays the same level. It seems that spectrum broadening exhibits some sort of stage character of “constant 1 - broadening - constant 2”. On the one hand, the laser operating regime depends on the net-cavity group-velocity dispersion (GVD), which is defined as the sum of each fiber’s GVD times its length^15^. The increase of spectral bandwidth of output laser can be attributed to dispersion compensation in the whole Raman laser system since the zero-dispersion wavelength of UHNA7 is 2.6 μm^6^. The narrow output spectrum is further considered to be achieved by better control of higher dispersion of the cavity, especially the third order dispersion (TOD), since the TOD of the UHNA7 fiber is large and negative and has normal GVD as well^16^.

A slightly red-shift of central wavelength from 2117.2 to 2118.6 nm is also noticed in Fig. 4, and the inset details the wavelength of Raman peak. Although typical two-peak structure of the Raman gain exists in silica fibers, the Raman shift is usually over 1 THz. The slightly red-shift of 1^st^ Raman stokes shift in this work is only 0.1 THz at 2.1 μm. We believe that further frequency shift is limited by the transmittance of the FBGs. Moreover, a maximum value of ratio of Raman power to total output power is turned up at incident pump power of 21.2 W in Fig. 5, which is just corresponding to the inflection point of the increase of output Raman power. When pump power reaches a certain level, the growth rate of both Raman power and ratio of Raman power to total output power both begin to decrease. The two different regimes of the Raman power scaling indicate that nonlinear effect induced power dissipation occurs at higher powers. It should be noted that the inflection point on power growth curve is just consistent with the red-shift of peak in spectral curve. At low pump powers, the short-wavelength peak dominates the Raman spectrum. Once the pump power increases, the long-wavelength peak begins to dominate the Raman spectrum. It is worth mentioning that as pump and Raman laser power increase, the temperature of HR2 (in Fig. 1) varies from room temperature to 54 °C. The increasing temperature of HR2 may influence spectrum shift as well.

With the increase of pump power, Raman spectrum broadening and peak wavelength shift could be caused by FWM in the high nonlinear fiber. In 2 μm region, the GVD parameter *β*_2_ of UHNA7 is about 0.00014 fs^2^/nm^16^. Significant FWM can occur even if phase matching is not perfect to yield condition of *κ* = 0. Assuming the mismatching contribution of material dispersion Δ*k*_M_ dominates *κ*, the coherence length *L*_coh_ can be related to the frequency shift *Ω*_s_ by using *L*_coh_ = 2π/|Δ*κ*| and is given by

1 $${L}_{{\rm{coh}}}=\frac{2{\rm{\pi }}}{|{\rm{\Delta }}{k}_{{\rm{M}}}|}=\frac{2{\rm{\pi }}}{|{\beta }_{2}|{\Omega }_{{\rm{s}}}^{2}}.$$

Because the pump wavelength (1.94 μm) is not highly close to the zero-dispersion wavelength of the UHNA 7 fiber (2.6 μm), we can use Δ*k*_M_ ≈ *β*_2_*Ω*_s_^2^. And for frequency shifts *ν*_s_ = *Ω*_s_/2π of about 14 THz in this work, the coherence length is about 250 m which is larger than the UHNA7 fiber length of 26 m. The possible near phase matching could generate FWM. The FWM Stokes and anti-Stokes peaks are generated below Raman gain threshold in Fig. 6(a). The spectra bandwidth of residual pump light at 1.94 μm after Raman laser is much wider than that before Raman laser in Fig. 6(b). According to ref.^17^, at low powers, the Raman Stokes wave spectrum should broaden due to the pump wave fluctuations brought by nondegenerate FWM. However, the Raman Stokes wave spectral width is defined by the FBGs transmission instead of pump wave power^18^. The transmission spectral profiles of FBGs (LR2 and HR2) are shown in Fig. 7. The bandwidth (FWHM) of LR2 is 1.2 nm, which is corresponding to the Raman Stokes bandwidth in “constant 1” stage. And the bandwidth (FWHM) of HR2 is 3.3 nm, which is corresponding to the Raman Stokes bandwidth in “constant 2” stage. In other words, at low powers, the Raman Stokes spectrum width is limited to the LR2 bandwidth. When pump power increases, nondegenerate FWM broadens the width of Raman Stokes spectrum until to the bandwidth of HR2. It can be expected that as the pump power continues to increase, the spectrum of Raman Stokes will keep the same as 3.3 nm for a certain power level and then continue to expand. Only the main spectral broadening mechanism will be quasi-degenerate FWM between Raman Stokes wave longitudinal modes itself^18^.

The decrease of ratio of Raman power to total output power further indicates that the Raman gain becomes small and the SRS is suppressed by FWM as pump power increases^19^. The gain competition between FWM and SRS (central wavelength red-shift in Fig. 4) may also lead to efficiency decrease of output Raman power (slope efficiency decrease in Fig. 3). The suppression of SRS and broadening of first-order Stokes spectrum both decrease energy density in Raman fiber. Since the first-order Stokes light can be the pump source of the second-order Stokes light, low energy density of the first-order Stokes light increases the threshold of the second-order Stokes light. In Fig. 8, the 2^nd^ Stokes light is not detected at 4.2 W Raman output in this experiment, and the output power of the first-order Stokes light is only limited by pump power.

The laser output spectra with or without FBG at 4.2 W Raman power are shown in Fig. 8. It is illustrated that only 1^st^ Raman stokes light is stimulated even though the pump power threshold of Raman lasers is reduced by adding FBGs as resonant cavity^20^. The Raman gain (*g*_0_) at 2.1 μm is estimated by *α*_S_ = *g*_0_*P*_th_, where *α*_S_ is optical loss in the fiber at 2.1 μm and *P*_th_ is threshold of the pump power^8^. The *α*_S_ is experimentally measured as ~23 dB/km, while the *P*_th_ is 12.1 W as mentioned above, which leads to *g*_0_ of ~1.9 dB/(km · W). It is expected that the value of Raman gain can be increased by optimizing the length of Raman fiber and the reflectivity of LR FBG.

To keep this Raman oscillator operating stably, two-way MFAs are used to replace fusing points between Raman gain fiber and other fiber. As given in Fig. 9, the splice loss between the 10 μm and 2 μm core diameter fiber is as large as 2.57 dB, while the insert loss of MFA is only 0.23 dB. The great splice loss can cause serious thermal accumulation. The output power of 8 W decreased to 4.5 W because of one fusing point losses. As a result, the temperature of fiber coating rises to 80 °C. In the situation of using MFAs, there is barely any heating induced by the laser loss and the power fluctuations is measured to be less than 2% in 0.5 hour, which further illustrates that this Raman fiber oscillator has significant power scaling potential. The higher efficiency will be expected in the future when the MFAs locate outside the Raman cavity and the FBGs are written in the UHNA7 fiber directly. Since the polarization characteristic of the laser can noticeably decrease Raman threshold, using polarization maintained fiber should be another method to optimize this Raman fiber laser.

## Methods

The schematic diagram of Raman oscillator pumped by monolithic single-mode Tm-doped fiber at 1.94 μm is shown in Fig. 1. Two pump LDs (laser diodes) at 793 nm were launched into the 1.94 μm laser cavity via a (2 + 1) × 1 fiber combiner with 8° end surface fiber. A resonator was simply formed by HR1 (high reflectivity) and LR1 (low reflectivity) FBGs, whose central wavelengths both located at 1941.82 nm. The gain Tm-doped fiber had a pure Tm-doped silicate core of 10 μm diameter with cladding absorption of 3.00 dB/m at 793 nm. A cladding stripper (CPS in Fig. 1) was used to eliminate the redundant light in inner cladding in order to acquire good beam quality for Raman laser. The heat accumulation on HR2 had been effectively reduced as well.

The Raman oscillator was simply and reliably formed by a pair of FBGs made by 10/130 μm fiber (FUD4070, Nufern) as well, whose central wavelengths both are located at 2117.7 nm. The reflectivity and bandwidth of HR2 were 99.5% and 3.3 nm respectively, while the parameters of LR2 becomes 48% and 1.2 nm separately. The highly Ge-doped (58 wt.% Ge doping) Raman gain fiber used in this experiment is 26 m UHNA7 from Nufern with *λ*_c_ of 1.45 μm and NA of 0.41, which has a core diameter of 2.4 μm and a zero dispersion of ~2.6 μm^6^. Furthermore, to reduce Raman laser threshold, two MFAs were used to sharply decrease heat accumulation and splice loss between 2.4 μm and 10 μm of different fiber core diameter. It should be noted that the MFAs are non-directional with insertion loss as low as 0.15 dB while the splice loss between UHNA7 and FUD4070 is far larger than 0.3 dB (since splice loss between UHNA7 and SMF-28 is 0.2–0.3 dB^21^).

The output power was measured by thermal power meter through a 10° dichroic mirror with high transmission (>95%) at 2118 ± 25 nm and high reflectivity (>99%) at 1940 ± 10 nm. The temperature of 1.94 μm laser system was stabilized by a 20 °C water-cooling heat sink, while the Raman gain fiber was pumped without any heat dissipation. As a matter of fact, Raman fiber lasers have advantages in practice over rare-earth-doped fiber laser because of the unnecessary heat management.
